# Wind turbine condition monitoring dataset of Fraunhofer LBF

**DOI:** 10.1038/s41597-024-03934-5

**Published:** 2024-10-09

**Authors:** Atabak Mostafavi, Andreas Friedmann

**Affiliations:** 1 Fraunhofer Institute for Structural Durability and System Reliability LBF, Darmstadt, Hessen 64289 Germany; 2 Technische Universität Darmstadt, Department of Mechanical Engineering, Darmstadt, Hessen 64287 Germany

**Keywords:** Mechanical engineering, Energy supply and demand, Wind energy

## Abstract

Fraunhofer wind turbine dataset contains monitoring data from a 750 W wind turbine, including accelerometers and tachometer, to capture structural response, bearing vibrations and rotational velocity. Additionally, temperatures, wind speed and wind direction have been measured, while weather conditions have been acquired from selected sources. Various damage scenarios, including mass imbalance, and aerodynamic imbalance as well as damages on bearings’ outer race, inner race and roller element have been implemented. The availability of time series data makes the dataset well suited for both machine learning and signal processing-based condition monitoring applications. The availability of heterogeneous sensors has created a dataset particularly suited for information fusion, data fusion, multi-sensor approaches, and holistic monitoring. Experiments were conducted in real-world conditions outside of a controlled laboratory environment, thereby introducing challenges such as variable rotor speed, noise, overloads, and other environmental factors. Consequently, the dataset is qualified for tasks involving uncertainty quantification and signal pre-processing. This document will detail the test equipment, experimental procedures, simulated damage cases and measurement parameters.

## Background & Summary

The energy demand is projected to reach 10^19 J by 2025 if the current economic growth trend persists. This underscores the critical importance of sustainable energy growth, which is essential for ensuring an improvement in the quality of life while simultaneously protecting the planet^1^. Renewable energy sources, due to their insignificant carbon footprint and their inexhaustible availability, are proven to be effective and efficient to tackle the current environmental crisis^2^.

**Fig. 1:**
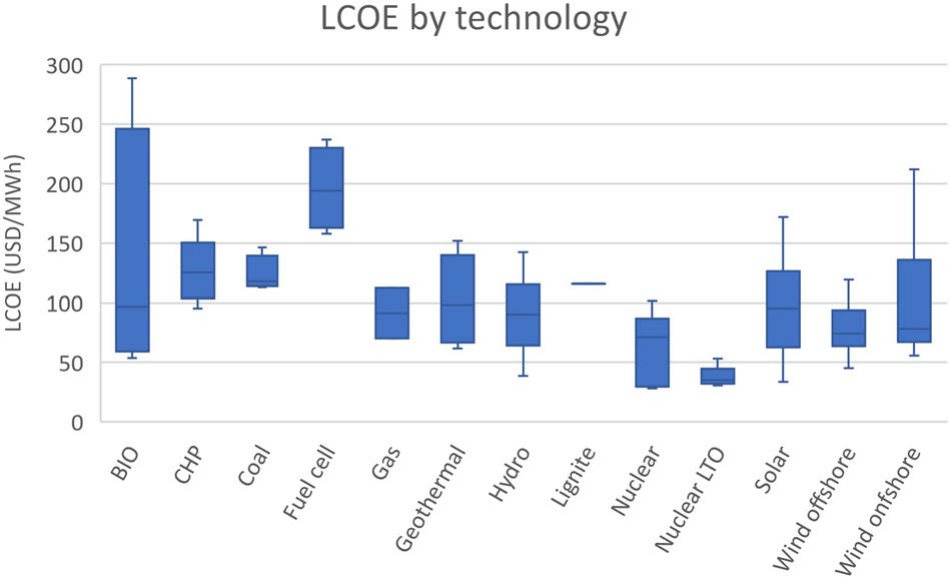
Levelized cost of energy by technology^3^.

Recent assessments from the International Energy Agency (IEA) indicate a noticeable shift towards the competitiveness of both onshore and offshore wind energy, in comparison to gas and emerging nuclear energy paradigms in Europe. Projections derived from contemporary analyses suggest that onshore wind energy will exhibit the lowest levelized costs of electricity generation of all sources by 2025. Moreover, the domain of offshore wind energy has undergone a notable evolution, emerging as a cost-competitive energy alternative. This has been precipitated by a substantial reduction in associated costs (e.g., production and maintenance) over recent years. Figure 1 illustrates the levelized cost of energy across various technologies as reported by the IEA for the year 2020. The data reveals that wind turbines (WT) already demonstrate a competitive levelized cost compared to other energy production technologies^3^.

### Condition monitoring

WT condition monitoring (CM) involves the systematic observation and analysis of operational parameters of a WT system or its essential components, aimed at detecting deviations from normal behaviour or indications of forthcoming faults. By observing changes in fault-indicating parameters, the process enables fault diagnosis (detection, isolation, and identification) and the estimation of remaining useful life before a potential failure or critical malfunction arises in the WT. CM facilitates a cost-effective and optimal mode of maintenance known as “condition-based maintenance”, wherein interventions are guided by observed conditions rather than adhering to fixed service intervals, thus, minimizing maintenance expenses by minimizing the number of unnecessary maintenance interventions. Additionally, since failures in various components of wind turbines, including sensors, actuators, rotor blades, generators, gearboxes, bearings, electrical systems, and electronic control units, can occur unexpectedly, CM helps with timely detection of these failures, facilitating the planning of maintenance activities, the procurement of necessary parts, and the mitigation of damage to other components^4^.

**Fig. 2:**
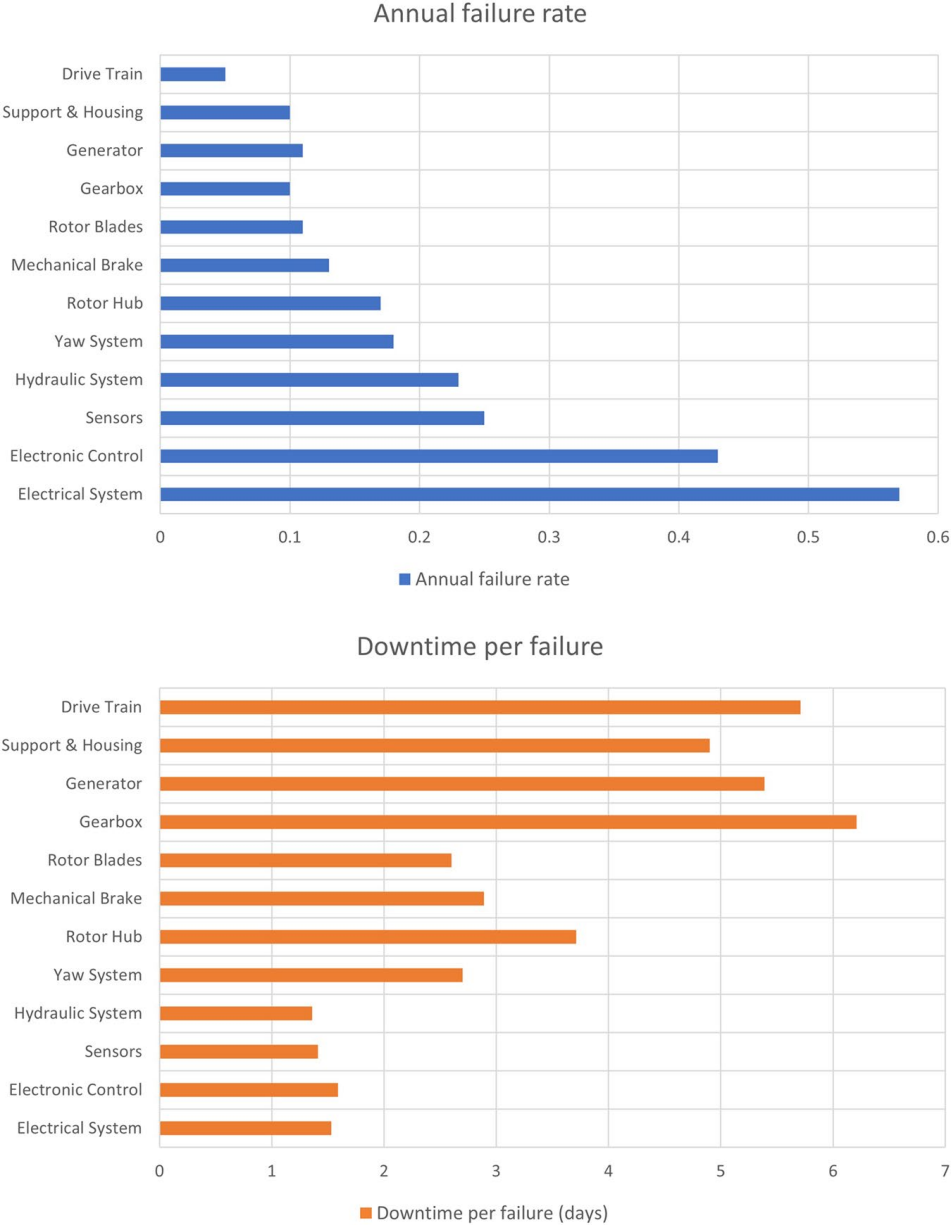
(**a**) Top: failure rate (**b**) Bottom: downtime^27^.

Numerous studies have explored potential damage scenarios in WT and their interrelationships with other forms of damage. Mechanical failures, particularly in bearings, gearboxes, and rotor aerodynamics/mass imbalance, are known for their frequent occurrence and resultant downtime. Despite the highest failure rates being attributed to the electrical system and controls, the associated WT downtime is relatively low. As depicted in Fig. 2, the primary sources of downtime are associated with issues in the drivetrain and critical rotating components, such as the rotor, gearbox, and generator, with bearings being a common element among them. Notably, the downtime linked to these components surpasses that of all other subassemblies, due to the complicated repair procedures. Transferring those results from onshore WT to offshore settings, it has to be noted those activities necessitates specialized lifting equipment like cranes and vessels, along with considerations for variable weather conditions, which can further complicate repair attempts^5^.

Over time, significant advancements in the field of CM have yielded a variety of technologies tailored to meet multiple monitoring requirements across different industries. Among these technologies, vibration analysis emerges as the preferred approach due to its exceptional efficacy in detecting faults at an early stage. Accelerometers, serving as the sensor of choice for capturing vibration signals, are favoured for their cost-effectiveness and user-friendly design, facilitating seamless monitoring and diagnosis of machinery health.

### Standards and regulation

Several standards offer guidance for machinery CM. For instance, ISO 13379^6,7^ covers data interpretation and diagnostics techniques, while ISO 17359^8^ provides general guidelines for the development of CM systems. DIN ISO 13373^9–12^ focuses on CM and diagnostics using vibration signals. Moreover, specialized standards address wind turbine CM, e.g., VDI 3834^13,14^, which delves into the measurement and assessment of mechanical vibration in wind turbines and their components. These standards play a crucial role in ensuring effective and reliable CM practices across various industries. While they offer recommendations rather than mandatory regulations, they provide valuable insights into possible approaches and solutions.

### Available datasets

Open datasets are valuable resources for researchers and experts that develop new technologies. Moreover, they foster collaboration and knowledge sharing within the research community, facilitating benchmarking and comparative studies across different monitoring techniques and industrial sectors. Incorporating open datasets in research work promotes transparency, reproducibility, and innovation. Here, CM is no exception, since with moving toward artificial intelligence and machine learning tools, the necessity of having open datasets available is increasing. Currently, several datasets suitable for the development of CM are available. The following few are highlighted here and summarized in the Table 1.

1. The Bearing dataset of IEEE includes different damage scenarios such as faults of inner race, outer race and rolling element, machined with different depths. Its Author claims, that dataset has obvious fault characteristics^15^.
2. The Prognostics Data Repository of NASA provides valuable datasets for various areas of CM including experimental bearing dataset, which contains accelerometers signals of four different damaged bearings^16^.
3. The Case Western Reserve University (CWRU) bearing dataset and the IEEE Gearbox datasets are two of the most commonly used datasets in the field of CM^17^.
4. The Machinery Fault Database (MAFAULDA) provides another bearing fault diagnosis dataset where data has been measured by accelerometers and microphones at variable rotor speed. Various faults such as imbalance, misalignment and bearing fault have been recorded using the SpectraQuest test bench with different level of severity^18^.
5. National Renewable Energy Laboratory (NREL) collected vibration signal from a healthy and a damaged gearbox tested by the GRC, and released the dataset together with their fault condition to offer a benchmark for CM community^19^.
6. A recent dataset from five Fuhrländer FL2500 2.5 MW WT has been published, containing certain statistical features (minimum, maximum, mean, and standard deviation) extracted in five-minute intervals from vibration signals. The dataset can be utilized for bearing and gearbox monitoring^20^.
7. The blade monitoring dataset from Mustanisiryah University contains vibration signals collected from a laboratory wind turbine test bench that simulates various blade faults, including erosion, mass imbalance, and blade twist^21^.

**Table 1:** Summary of available datasets for CM.

Dataset	Source	Description	Fault Types/Scenarios	Notes
Bearing Dataset of IEEE	IEEE	Damage scenarios machined with different depths	Bearing faults	Evident fault signature
Prognostics Data Repository of NASA	NASA	acceleration signals from four different damaged bearings.	Bearing faults	Valuable resource for various CM applications.
Case Western Reserve University (CWRU) Bearing Dataset	CWRU	Multiple damage size generated by EDM	Bearing faults, gearbox faults	Widely used in the field of CM.
Machinery Fault Database (MAFAULDA)	SMT	Acceleration and acoustic signal Generated by SpectraQuest Test Bench	Imbalance, misalignment, bearing faults	Data collected at different levels of severity and rotational speed.
National Renewable Energy Laboratory Gearbox Dataset	NREL	Vibration signals from a healthy and damaged gearbox tested by GRC.	Gearbox faults	Released with fault condition information for benchmarking.
Fuhrländer FL2500 2.5 MW WT Dataset	Fuhrländer	statistical features extracted from vibration signals	Bearing and gearbox faults	Recorded from operational WT
Blade Monitoring Dataset from Mustanisiryah University	Mustanisiryah University	vibration signals from scaled wind turbine	Blade erosion, mass imbalance, blade twist	Focused on blade fault diagnosis in wind turbines.

Datasets dedicated to the CM of machinery are not limited to the mentioned examples; however, the available data for WT monitoring is limited, and if available beyond above mentioned sets, they contain discrete features which limits their applicability for signal processing studies or advanced deep learning algorithms such as convolutional neural networks. Furthermore, the majority of datasets originate from designed test benches where damage is readily identifiable, and the quality of data is not comparable to real-world scenarios. Consequently, an attempt has been made to create a dataset for CM of WT that closely resembles real-world conditions. This includes the effects of the surrounding environment, data challenges and the highly volatile nature of wind speed.

### Novelty of fraunhofer dataset

The contribution of the work to the choice of open datasets can be summarized as follows:

- operation of a wind turbine under open-air conditions with variable wind speeds and thus varying rotational speeds
- providing an aerodynamic mic imbalance fault dataset to study pitch misalignment faults in wind turbines
- providing a more realistic dataset by utilizing a scaled commercial wind turbine instead of laboratory test benches
- providing a heterogeneous multisensory dataset to understand the effect of damage on the different parts of the structure, thus enabling studies on information fusion to enhance system diagnosis
- Providing time series and not only extracted statistical or vibrational features, will enable researchers and scientists from the fields of signal processing and machine learning to evaluate their sophisticated technologies.

Possible use case of the dataset can be named as but not limited to: a. information fusion application to enhance CM systems using various available information, b. study the effect of damage in the whole system e.g., detecting imbalance with foundation sensors, c. holistic monitoring, d. studying pitch control algorithms using aerodynamic imbalance cases, e. icing or blade damage scenario using mass imbalance cases f. bearing monitoring under highly volatile and low rotational speeds.

### Limitation of fraunhofer dataset

Although the selected scaled specimen facilitated the execution of the measurement campaign, the direct-drive WT overlooks the necessity for gearbox condition monitoring studies.

### Future improvement

The output power of the WT holds valuable information regarding its performance and condition. Including voltage and current of WT in a condition monitoring dataset would be a significant enhancement for future work.

### Previous studies

**Fig. 3:**
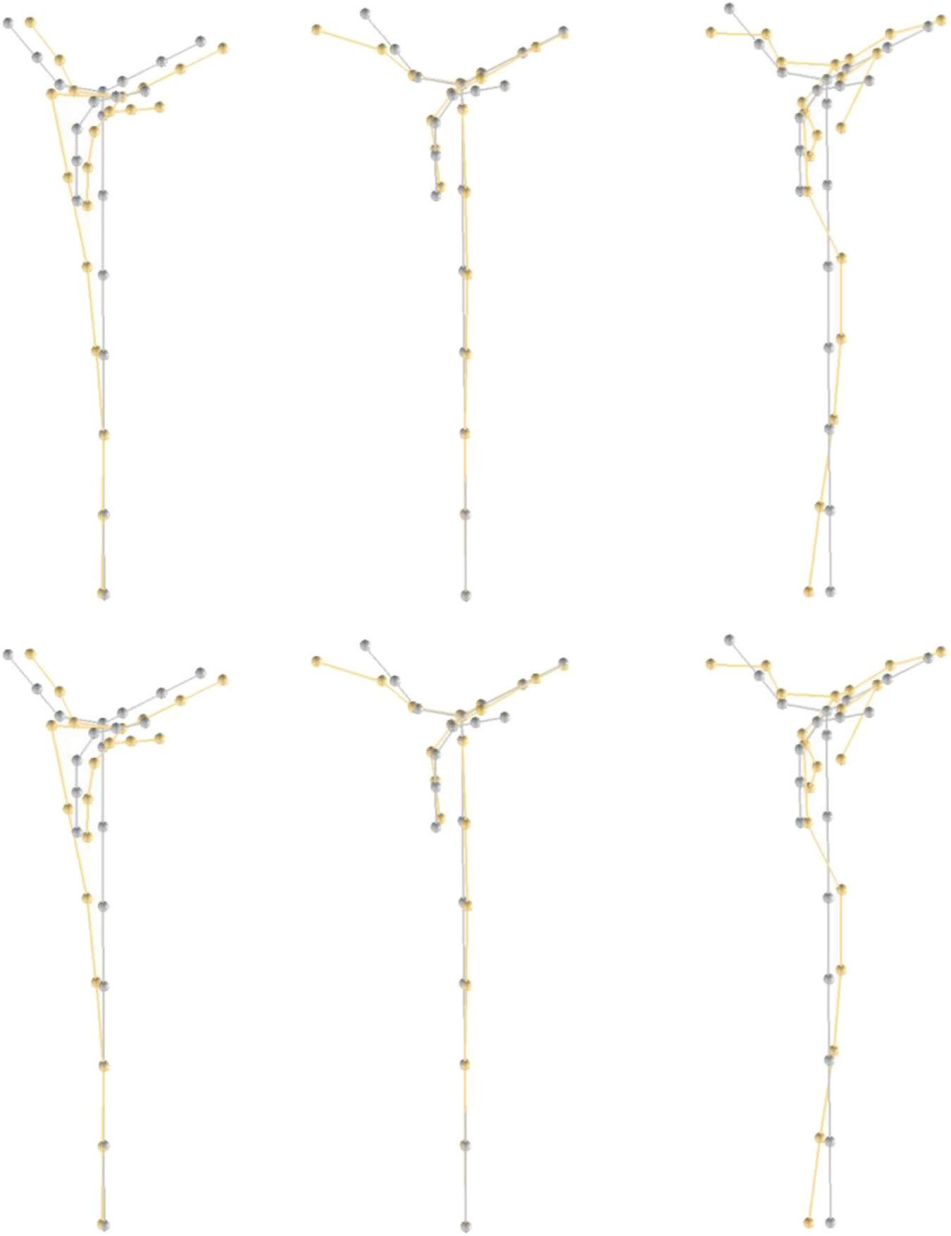
Selected modes of the experimental modal analysis. (**a**) Top row, left to right: Mode 1 @2.79 Hz Mode 5 @20.89 Hz Mode 11 @47.18 Hz (**b**) Bottom row, left to right: Mode 2 @2.84 Hz Mode 6 @23.83 Hz Mode 12 @53.21 Hz^22^.

Previously, a separate author conducted a numerical and experimental structural modal analysis on the same structure in a separate study. This analysis sheds light on the behaviour of the structure itself and offers information about the mode shapes and fundamental frequencies. Figure 3 depicts the six most important mode shapes as found within the study. Table 2 shows a list of the first five fundamental frequencies. For a more detailed explanation, please refer to the reference document^22^.

**Table 2:** Results of the experimental modal analysis^22^.

Mode	Frequency	Damping
1	2.79	0.53%
2	2.84	0.36%
3	15.12	4.54%
4	19.08	0.87%
5	20.89	1.33%

## Methods

A detailed description of the used instrumentation during the measurement process, the sensor positioning, the measurement parameters, the characteristics of the test setup, the test environment, and the damage introduced is provided herein.

### Technical details of WT

An AeroCraft AC-752 series 750 W direct-drive WT with a 12-pole permanent magnet generator and 2.4 m blade diameter has been installed on the roof of a two-story building in Darmstadt, Germany. The turbine is mounted on a 7 m mast, bringing the total hub height to 14.5 m above ground. The rotor shaft is supported by two roller bearings, one between hub and generator, which carries the majority of the load, and the other on the other side of the generator. Detailed technical specifications of the WT are given in Table 3 and additional information can be found in the manufacturer’s technical data sheet^23^. The entire structure is mounted on a pivot, allowing the WT to be easily lowered and raised for sensor installation or setup modifications. The tower is attached to the support wall at two different points using brackets.

**Table 3:** Technical specifications of WT^23^.

No.	characteristic	value
1.	permanent magnet generator	12 poles
2.	nominal output power	750 W
3.	nominal wind speed approx.	9 m/s
4.	cut-in wind speed approx.	3 m/s
5.	critical wind speed	40 m/s
6.	nominal rotational speed	600 rpm
7.	number of rotor blades	3
8.	material of rotor blades	glass-fiber reinforced plastic
9.	diameter of rotor blades	2.4 m
10.	swept area of rotor	4.5 m²
11.	azimuth control	wind vane
12.	pitch angel	fixed to 9°

### Sensors

**Fig. 4:**
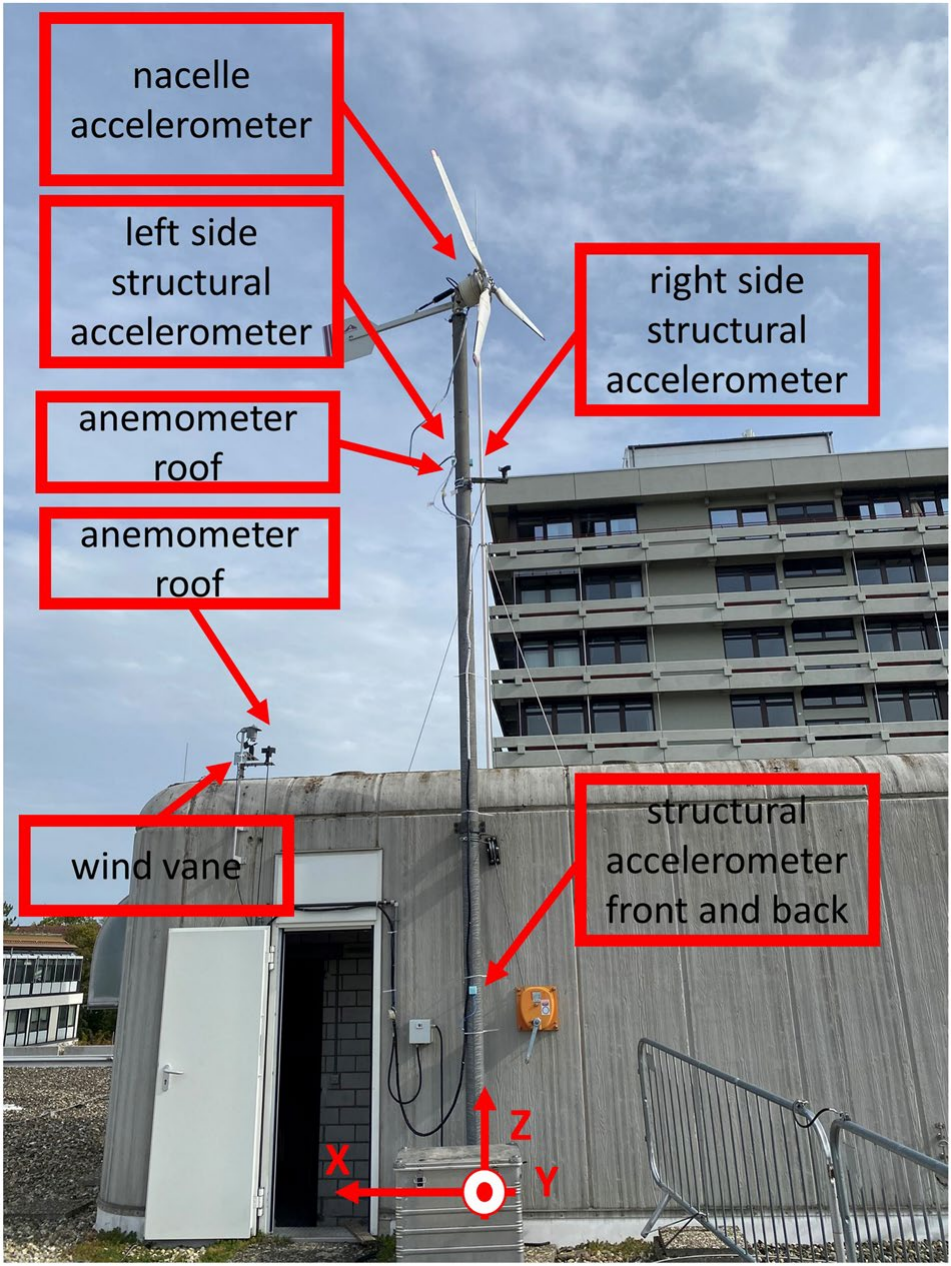
WT and sensors position.

**Fig. 5:**
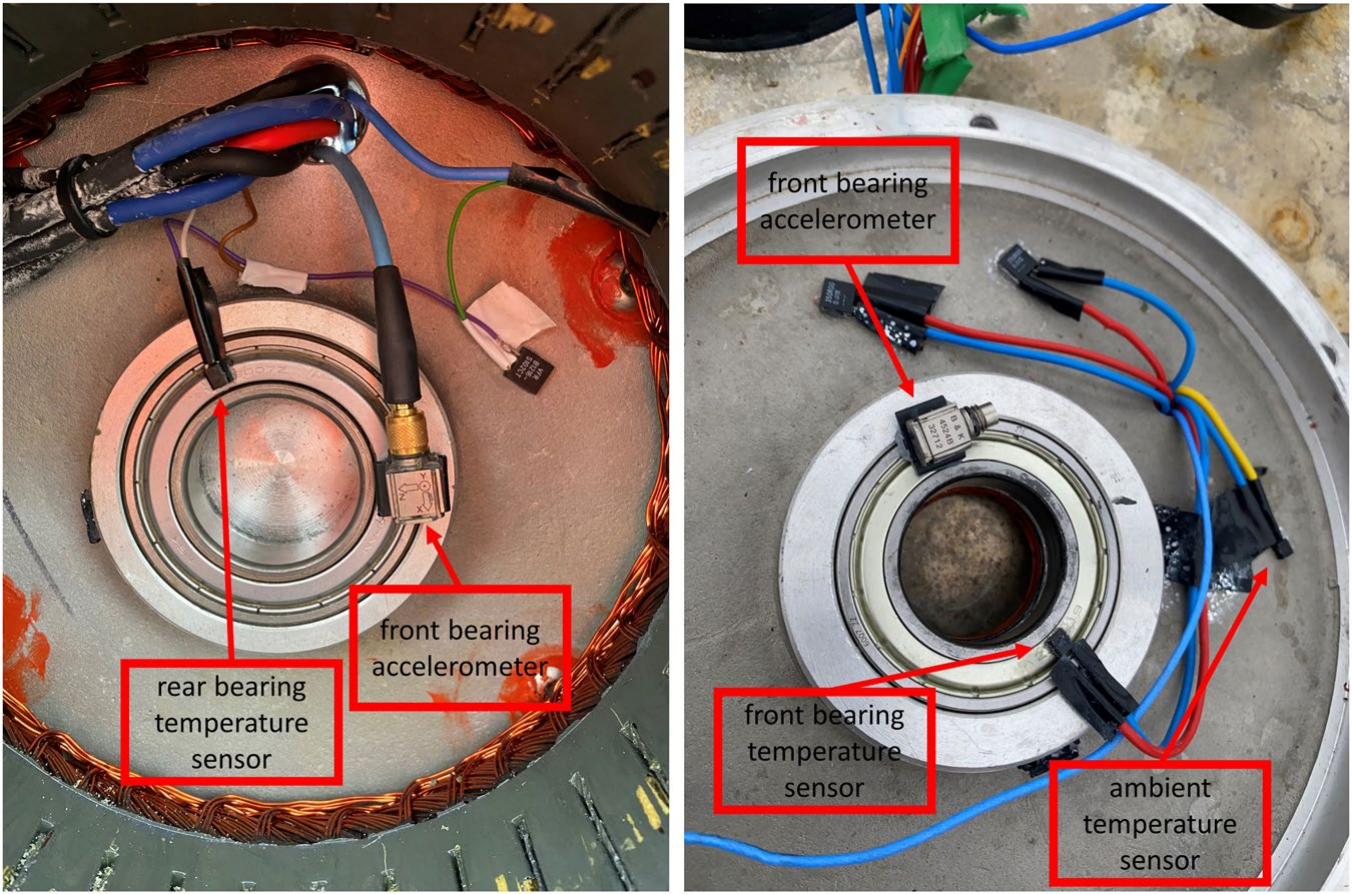
(**a**) Left: rear bearing and sensor position (**b**) Right: front bearing and sensor position.

Vibration measurements were conducted using seven Brüel & Kjaer type 4524 triaxial accelerometers. Two accelerometers were placed at the bottom of the structure, between the first and second bracket, 180 degree apart. Two additional accelerometers were installed above the brackets, positioned out of the plane from the previous two, and as well spaced 180 degrees apart. The fifth accelerometer was mounted on top of the nacelle, as depicted in Fig. 4. The sixth and the seventh accelerometer were installed at the front and rear bearing, respectively, as illustrated in Fig. 5. Accelerometers are capable of measuring within the frequency range of 0.2 to 5000 Hz with an error margin of less than 10%, while maintaining a nominal sensitivity of 100 mV/g up to 50 g.

**Fig. 6:**
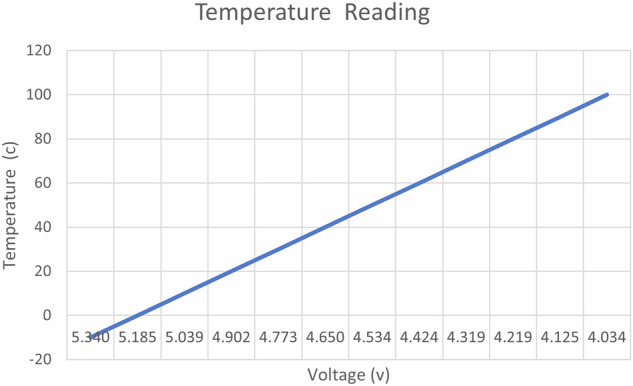
Temperature reading from voltage output of PT-1000 sensor.

Two PT-1000 temperature sensors were mounted on each bearing, along with an additional PT-1000 sensor positioned inside the nacelle, as shown in Fig. 5. These sensors were used to monitor the temperature of each bearing and the ambient temperature within the nacelle. The PT sensors were installed in series with a resistor of 350 ohm and supplied with 20 volts as input. Voltage signals were subsequently measured from the known resistance, with an expected voltage of 5.18 volts at zero degrees. Figure 6 shows the temperature reading from the output voltage of these sensors.

**Fig. 7:**
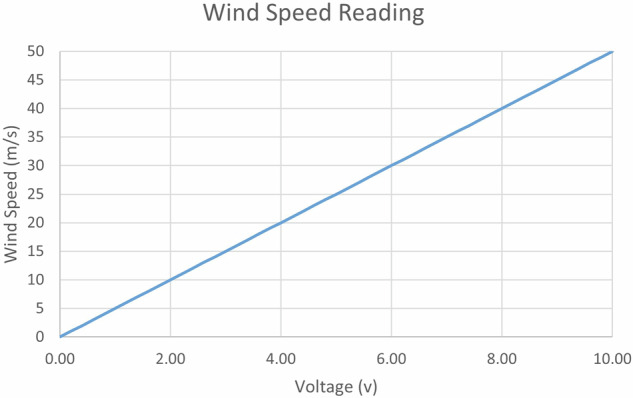
Wind speed Reading from anemometer.

Two anemometers have been placed at heights of 9 and 12.5 meters above ground level to measure wind speed, as depicted in Fig. 4 These anemometers, manufactured by PCE Instrument, are designed with an output range from 0 to 10 volts. Within this range, they are capable of measuring wind speeds ranging from 0 to 50 meters per second, as illustrated in Fig. 7.

**Fig. 8:**
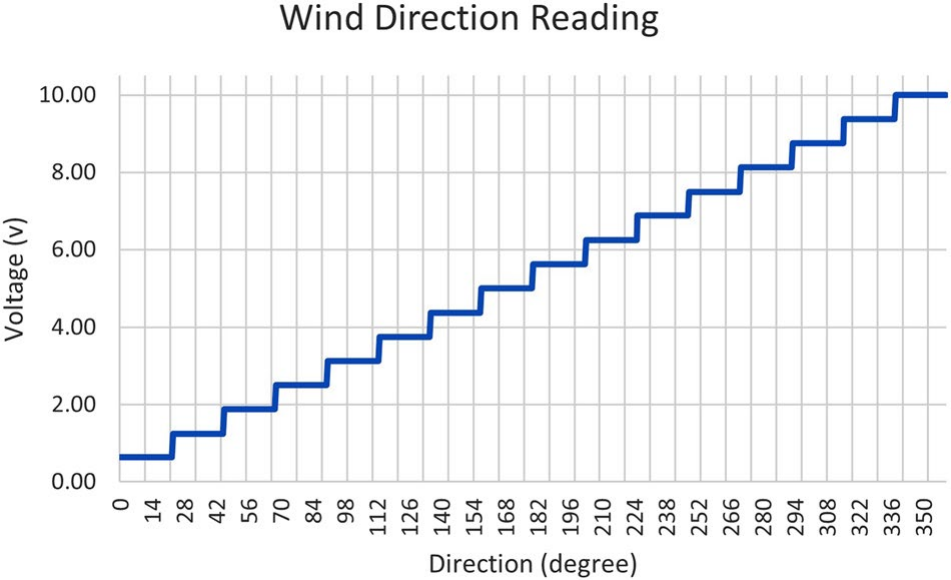
Wind direction reading from wind vane.

A wind vane, as shown in Fig. 4, has recorded wind direction with a resolution of 22.5 degrees. Each directional bin was represented by a specific voltage between 0 and 10 volts. With zero volts representing north at zero degrees and increasing in a clockwise direction, such that 2.5 volts indicates east at 90 degrees, and it returns to 0 volts representing north at 360 degrees. Figure 8 illustrates the interpretation of wind direction readings derived from the output voltage of the wind vane.

The rotational speed of the WT was measured using a hall effect tachometer equipped with 108 pulses per rotation.

### Measurement device

The measurement was conducted using a DEWE-501 model equipped with 64 channels, manufactured by Dewetron. The software employed for the measurement was Dewesoft V6.6.

### Environmental condition

Weather conditions, including temperature, wind speed, wind gust, and others, were documented in separate files sourced from the nearest weather station, situated in Kranichstein station, Darmstadt, Hesse, Germany^24^.

### Measurement parameters

To optimize data size, various sampling frequencies were selected for each instrument, taking into account their frequency range and the frequency range expected for the signals they record. This was implemented by dividing the original sampling frequency with the so-called sample rate divider capability of the Dewetron measurement device. The original sampling frequency was set to 74 kHz for accelerometers installed on the bearings. The sampling ratio for nacelle accelerometers was adjusted to 37 kHz by setting the sample rate divider to a 1/2 ratio. Structural accelerometers and the tachometer had their sampling ratios reduced to 2.95 kHz by setting the sample rate divider to 1/25. The remaining instruments utilized a sampling ratio of 1.48 kHz achieved by dividing the original sampling frequency by fifty. A summary of sensors’ specifications including the measurement parameters such as reference sensitivities, sampling frequencies, anti-aliasing filters, and measurement ranges are provided in the Table 4.

**Table 4:** sensor specification summery.

Sensor Type	Quantity	Placement	Specifications	Variable name in data file	Sampling Frequency (kHz)	Anti-Aliasing Filter (kHz)	Range (Volts)
Triaxial Accelerometers	1	Front bearing	Frequency range: 0.2 to 5000 Hz, Error margin: < 10%, Sensitivity: 100 mV/g up to 50 g	Brng_f_x, Brng_f_y, Brng_f_z,	74	30	−2.5 to +2.5
	1	Rear bearing		Brng_r_x, Brng_r_y, Brng_r_z,			
	1	Top of nacelle		Nacl_x, Nacl_y, Nacl_z	37	10	
	2	Top left and right of mast (above brackets, 180° apart)		top_l_x, top_l_y, top_l_z, top_r_x, top_r_y, top_r_z	2.95	1	
	2	Bottom front and rear of mast (between 1st and 2nd bracket, 180° apart)		bot_r_x, bot_r_y, bot_r_z, bot_f_x, bot_f_y, bot_f_z			
Temperature Sensors	2	One on each bearing	Installed with 350-ohm resistor, 20 V input, 5.18 V at 0 °C	tmp_brng_f, tmp_brng_r	1.48	—	0 to 10
	1	Inside the nacelle		tmp_amb		—	
Anemometers	2	9 m and 12.5 m above ground level	Output range: 0 to 10 V, Wind speed: 0 to 50 m/s	anm_roof, anm_mst		—	
Wind Vane	1	9 m above ground	Resolution: 22.5°, Voltage: 0 to 10 V, 0 V = North (0°), 2.5 V = East (90°)	van		—	
Tachometer	1	Mounted on WT	108 pulses per rotation	tach	2.95	1	

### Damage scenarios

**Fig. 9:**
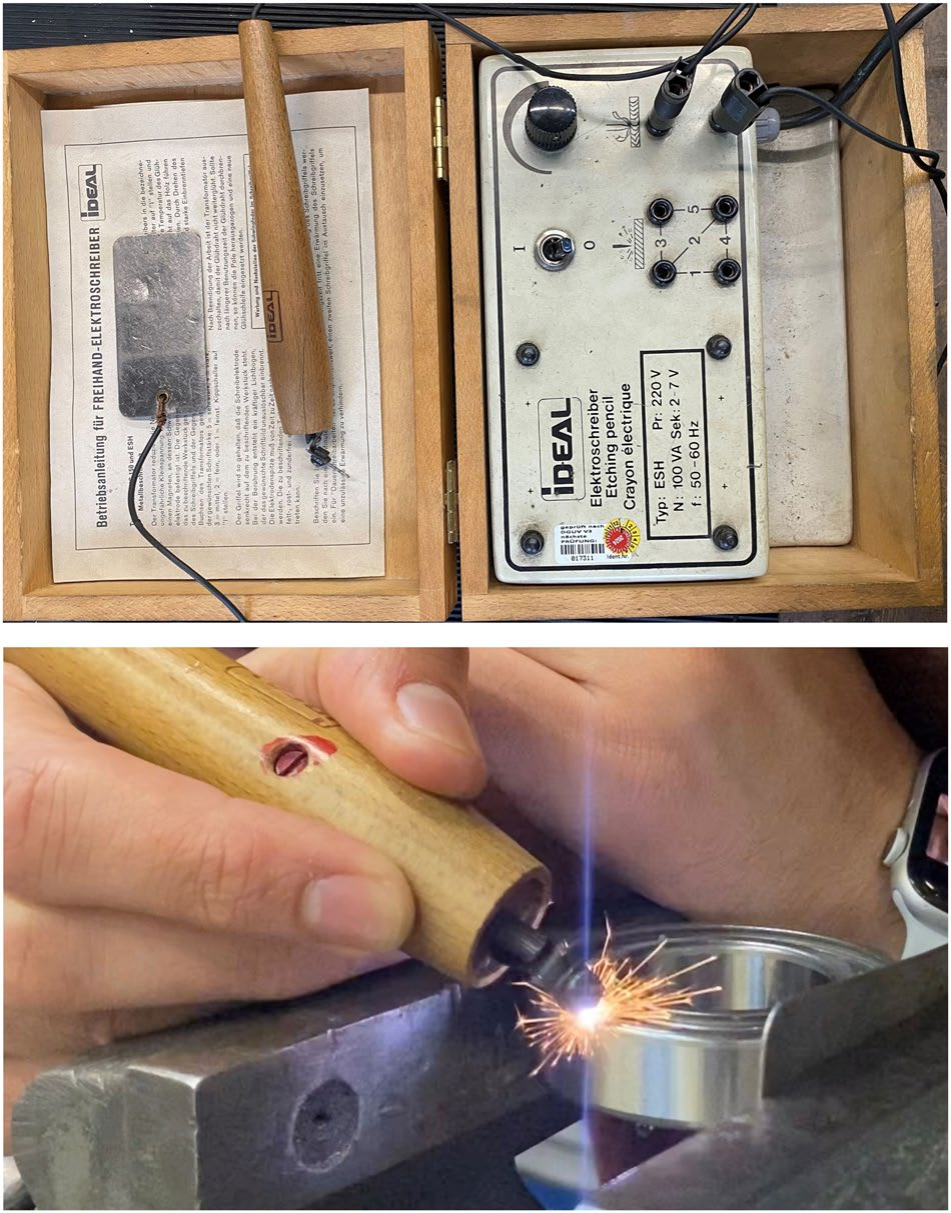
(**a**) Top: IDEAL etching pencil (**b**) Bottom: etching outer race of bearing.
- A complex damage scenario was created by simultaneously inducing mass imbalance, centred at 33 cm from the rotational axis, and inner race damage on the bearing. This combined scenario is designed to facilitate the study of fault correlation and the identification of individual damage among multiple dynamic factors, providing a more challenging and realistic situation for analysis and diagnosis.

Data from the healthy working condition, serving as the baseline, and four categories of damages manually generated on the structure have been recorded. A summary of the damage scenarios and their respective measurement durations is provided in Table 5. A description of each damage case follows:

- Mass imbalance was induced by adding a 170 g rigid mass to one blade to simulate an imbalance scenario. This test was conducted three times with the imbalance mass centred at 33 cm, 58 cm, and 73 cm away from the rotational axis to simulate different degrees of damage severity. This scenario serves as case study for the development of icing and blade damage diagnostic systems.
- Aerodynamic imbalance was induced by manually adjusting the pitch angle of one blade, while maintaining the pitch angle of the other blades at a fixed value. This test was repeated three times, each time modifying pitch angles by 5, 12, and 15 degrees, respectively, to generate varying levels of severity. This scenario is valuable for studying pitch misalignment and its effects on wind turbine performance.
- Three types of bearing damage, including inner race, outer race, and roller element, were simulated using an IDEAL etching pencil. This tool is suitable for scratching metal surfaces, as depicted in Fig. 9(a,b). This scenario serves as a valuable tool for studying bearing CM techniques and evaluating their effectiveness in detecting and diagnosing various types of bearing failure.

**Table 5:** List of damage cases together with their severity levels and measurement durations.

No.	case	severities	duration (min)
1	mass imbalance	3	3*60
2	aerodynamic imbalance	3	3*60
3	bearing outer race fault	1	20
5	bearing inner race fault	1	60
6	bearing inner race fault + mass imbalance	1	55
7	bearing roller element fault	1	60
8	healthy condition	—	90

### Bearing characteristics

Two identical deep groove ball bearings, specifically the 6007 2Z model manufactured by UBC, support the rotor shaft of the wind turbine. These bearings have a radial bore diameter of 35 mm, an outer diameter of 62 mm, a maximum speed rating of 11000 rpm, and are equipped with 11 balls with a diameter of 8 mm. With the provided information and using the fundamental bearing equations^25^ the bearing fundamental frequencies can be calculated as follows:

- Ball Pass Frequency Outer (BPFO) = 4.593 * f Hz
- Ball Pass Frequency Inner (BPFI) = 6.407 * f Hz
- Ball Spin Frequency (BSF) = 5.995 * f Hz
- Fundamental Train Frequency (FTF) = 0.417 * f Hz

Here, “f” represents the rotational velocity in Hertz.

### Boundary conditions

The WT structure was affected by the lowering and lifting processes facilitated by a manual winch for instrumentation and fault simulation purposes. These processes involved opening and closing the bracket fixtures, and in some cases, disassembling the WT itself to access the bearings. Such activities are similar to maintenance operations on the structure and are expected to influence its boundary conditions.

## Data Records

The data was continuously recorded and stored in “.mat” files in 5-minute intervals. However, if the wind speed during the measurement fell below the cut-in speed, the stored file was deleted to minimize the required storage capacity. Consequently, the length of the data files for the last measurement in each category might vary, due to early stoppage. The amount of useful data available for each scenario differs based on environmental conditions and the availability of the test bench.

The measurement files are named according to the fault condition, date, and time of measurement, following the format described [Fault Condition][Date][Time]. For example, a file named “ InnerRace_2023_12_11_143445” indicates a measurement acquired on December 11, 2023, at 14:35:45 for the inner race damage condition.

Measurements have been organized into distinct folders based on their fault condition, allowing for separate access and is publicly available on Zenodo through^26^: 10.5281/zenodo.11820597.

## Technical Validation

The experiments have been designed given consideration to the previously introduced standards. Measurement parameters and sensor position have been selected given consideration to IEC 61400, chapter 6, section 4 and VDI 3834 part one, chapter 3. Measurement condition and duration has been defined considering IEC 61400, chapter 6, section 3 and VDI 3834 part one, chapter 2, subsection 3. Data validation has been conducted by signal processing, feature engineering and utilizing a vanilla machine learning algorithm to ensure the compliance of data with all available tools in CM technology.

### Signal processing validation

**Fig. 10:**
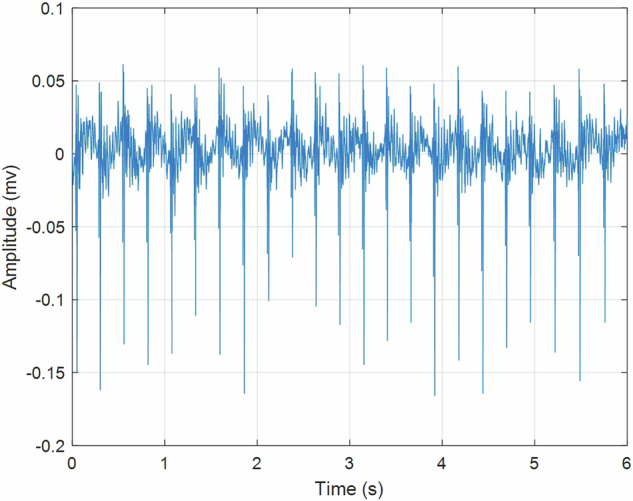
Front bearing x-direction accelerometer time signal in outer race fault condition (file: OuterRace_2023_12_20_183828, second 114 to 120).

**Fig. 11:**
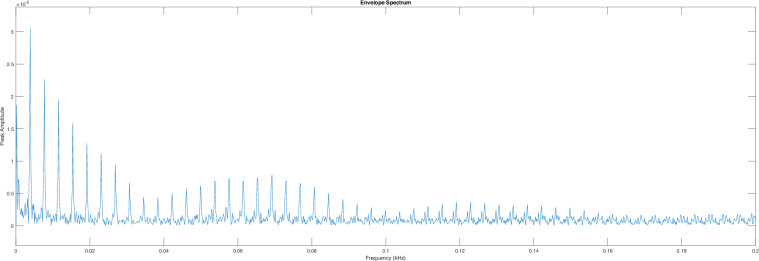
Hilbert envelope spectrum of filtered signal in range of 700 to 3000 Hz average RPM 50, BPFO 3.8 Hz.

Six-second intervals were extracted from the data, as depicted in Fig. 10. Subsequently, kurtograms were employed to determine the appropriate filter size. Following the application of the filter, Hilbert envelope analysis was conducted to verify the identifiability of bearing damage. As illustration, one scenario with outer race damage has been depicted in Fig. 11. There, the pick height at BPFO indicate the existence of damage.

### Feature engineering

In this verification technique, both faulty and healthy signals were segmented into multiple shorter duration signals and analyzed using MATLAB’s Feature Designer app to extract statistical features of the signals that are capable of distinguishing between healthy and faulty conditions. For instance, features have been extracted from high-severity mass imbalance faulty data obtained from several six-second segmented signals with rotational speeds between 20 to 30 RPM and listed in Table 6. High T-test values between healthy and faulty conditions indicate the distinguishability of these two groups.

**Table 6:** Mass imbalance data validation via feature engineering.

No.	Feature	T-Test
1	Kurtosis	29.0
2	Clearance Factor	27.9
3	Impulse Factor	27.7
4	Crest Factor	22.7
5	Skewness	16.6
6	Shape Factor	16.2
7	Std	9.2
8	RMS	9.2

## Usage Notes

For the purpose of reading and analyzing the provided dataset, both MATLAB and Python are recommended. Given that the dataset is affected by 50 kHz electrical noise, it is advisable to apply a suitable filter, such as a Butterworth bandstop filter, in order to enhance the signal-to-noise ratio (SNR). Furthermore, in order to convert the tachometer signal to revolutions per minute (RPM), it is recommended to use thresholds of 1.95 V and 2.05 V for the detection of the signal’s rise and fall, respectively.

## Data Availability

No custom code was utilized in this study. Data validation was conducted using standard MATLAB functions and toolboxes.
